# No Evidence of Responding Individuals Constraining the Evolution of the Pheromone Signal in the Pine Engraver *Ips avulsus*

**DOI:** 10.1007/s10886-022-01396-w

**Published:** 2022-12-10

**Authors:** Joséphine Queffelec, Brian Sullivan, Jessica L Mckenney, Jeremy D Allison

**Affiliations:** 1 Canadian Forest Service, Natural Resources Canada, Great Lakes Forestry Centre, 1219 Queen Street East, P6A2E5, Sault Ste. Marie, ON Canada; 2 U.S. Forest Service, USDA, Southern Research Station, Pineville, USA; 3 Department of Entomology, Louisiana State University, Baton Rouge, USA; 4 Department of Zoology and Entomology, University of Pretoria, Pretoria, South Africa; 5 Forestry and Agricultural Biotechnology Institute, Pretoria, South Africa

**Keywords:** Bark beetle, Enantiomeric ratio, Asymmetric tracking, Ipsdienol, Ipsenol

## Abstract

Chemical signals are important mediators of interactions within forest ecosystems. In insects, pheromone signals mediate intraspecific interactions such as mate location and acceptance. The evolution of pheromones in insects has been mostly studied from a theoretical perspective in the Lepidoptera. With this study, we aimed to broaden our understanding of pheromone communication in bark beetles. We first demonstrated that the enantiomeric ratios of ipsdienol produced by male *I. avulsus*, showed little variation. Subsequently, with field trapping trials we characterized the influence of the enantiomeric ratio of ipsdienol (pheromone component of *I. avulsus*) on *I. avulsus* captures and observed a great amount of variation in the receiver preference function. Most importantly, we demonstrated that responding individuals responded indiscriminately to all the enantiomeric ratios produced by the emitting individuals. These observations are consistent with the asymmetric tracking model which postulates that if the limiting sex is the emitting sex, responding individuals should not discriminate between emitted ratios. Consequently, responding individuals do not constrain the evolution of the signal. Our data suggest that, in *I. avulsus*, the composition of the aggregation pheromone signal might be more responsive to external selection forces, such as predation and metabolic constraints, as suggested by the asymmetric tracking model.

**Supplementary Information:**

The online version contains supplementary material available at 10.1007/s10886-022-01396-w.

## Introduction

Olfaction differs from other sensory modalities through a high degree of specificity. This specificity is possible through the necessary presence of a compound-specific receptor in the receiver. There is evidence of genetic independence between signaling and responding traits within some species of insects (Löfstedt et al. 1989; Allison et al. 2008), indicating that signal and response phenotypes must co-evolve in these species. Coevolution is possible if at least one of them exerts selective pressures on the other to either maintain or change the character state (Allison and Cardé 2016). This is illustrated in the bark beetle *Ips pini* where disruptive selection through preferential mate choice towards rare pheromone blends by females maintains diversity in pheromone blends within the male population (Shumate et al. 2011).

Several models have been proposed to understand the evolution of pheromone blends in the Lepidoptera (Allison and Cardé 2016). These models differ in the selective forces proposed to act on signalers and receivers. Although the chemical ecology of the Lepidoptera has been well studied, empirical data for testing predictions of these models is limited. Predictions of these models may be validated by comparing receiver preference functions to the distribution of the pheromone signal within signaling individuals. Variance ratios between emitting and receiving individuals should dictate the strength and type of selection acting on populations and their pheromone blends. In this study we aim to broaden the dialogue about pheromone evolution to include bark beetles (Coleoptera: Curculionidae: Scolytinae).

Pheromone-based tactics are important components of integrated pest management programs for many forest insect pests (Stenberg 2017). By decreasing the lifespan or disrupting mating of pest insects these tactics can impose selective pressures on the olfactory communication system between conspecifics of the target species. The potential for these selective pressures to result in the evolution of resistance in the pest population is under debate. So far, only one study has reported a phenomenon consistent with pheromone-based integrated pest management tactics selecting for change in pheromone signal traits (Mochizuki et al. 2002). Understanding the many evolutionary forces that affect pheromone blends could help improve pheromone-based tactics and our understanding of forest pest ecology (Allison and Cardé 2016).

*Ips avulsus* (Eichhoff) (Coleoptera: Curculionidae: Scolytinae) is an engraver bark beetle present in North and Central America and the Caribbean Islands and can be a significant pests of *Pinus* spp. (Connor and Wilkinson 1983). Most of the life cycle of this beetle occurs under the bark of host trees where larvae feed on phloem tissue. Adult males emerge and disperse to locate new, susceptible hosts, possibly by using host volatiles. After entry into host tissues, males release a pheromone that females and other males use to locate the host, often resulting in large aggregations (Connor and Wilkinson 1983). Males mate with one or multiple females within one host, sometimes helping with mining galleries for oviposition (Cook et al. 1983). Adults commonly reemerge to locate new resource and produce additional brood (Flamm et al. 1987). Ipsdienol, a pheromone component that occurs widely in the genus *Ips*, has been identified as a major component of the pheromone blend produced by male *I. avulsus.* The enantiospecific production and attraction of this compound is population specific (Smith et al. 1990; Kohnle et al. 1994; Strom et al. 2003; Miller and Allison 2011).

The evolution of pheromones in bark beetles has received limited attention (Symonds and Elgar 2004; Allison and Cardé 2016; Symonds and Gitau-Clarke 2016). In this study we investigate the evolutionary processes that act on the pheromone composition in *I. avulsus*. We first identify the enantiomeric ratios of ipsdienol that capture the most *I. avulsus* beetles (Allison et al. 2012) and characterize signal variation among male *I. avulsus* in terms of ipsdienol enantiomeric ratios. By comparing the distribution of the pheromone signal within signaling individuals to the receiver preference function, we observe that signal and response traits in *I. avulsus* are consistent with the asymmetric tracking model of pheromone evolution (Phelan 1992). Finally, we discuss the ecological traits and selective forces unique to bark beetles that could lead to the observed relationship between signalers and receivers.

## Methods and Materials

*Characterization of* I. avulsus *Preference Function.* Two trapping experiments took place in the Kisatchie National Forest, Catahoula Ranger District, Louisiana, USA. These experiments were conducted between 2010 and 2011 using eight-unit multiple funnel traps (ConTech Enterprises Inc.) equipped with a wet collection cup filled ca. one-third with propylene glycol. Trap surfaces were treated with PTFE (Northern Products Inc., Woonsocket, Rhode Island, USA) to increase trap captures (Allison et al. 2014, 2016). Within each experiment, traps were baited with three compounds, two that did not change among traps as well as one compound the enantiomeric ratio of which changed among traps. The compounds were chosen based on the literature (Allison et al. 2012). In both experiments each treatment (7 enantiomeric ratios) had 8 replicates with a total of 56 traps. All experiments were conducted in stands of *Pinus taeda* and mixed hardwoods that had experienced a prescribed burn one to two years before the experiments. Rope was tied between two trees and traps suspended individually with the collection cup ca. 1 m above ground level. Traps were at least 2 m from the nearest tree and 20 m apart within and among blocks. Bubble cap lures loaded with one of seven enantiomeric ratios of either ipsdienol or ipsenol and lanierone were purchased from Contech Enterprises Inc, Victoria, British Columbia, Canada. The chemical purity of all three pheromones was > 98% and release rates were 0.1–0.2 mg/d for ipsenol and ipsdienol, and 0.02 mg/d for lanierone (determined by the manufacturer at 25 °C). Traps were checked four times at two-week intervals. Beetles were identified using elytral armature (Wood 1982) and counted.

The first trapping experiment aimed to identify the preference function of *I. avulsus* in terms of ipsenol enantiomeric ratios. *Ips avulsus* does not produce ipsenol (Birgersson et al. 2012), however, this compound is produced by the sympatric bark beetle *Ips grandicollis* Eichoff and has a synergistic effect on *I. avulsus* response when combined with *I. avulsus* pheromone compounds (Allison et al. 2012). Different enantiomeric ratios of ipsenol might induce different behavioural responses from *I. avulsus*. This experiment took place June-August 2011 and all traps were baited with lanierone and racemic ipsdienol and one of seven enantiomeric ratios [(+):(-): 3:97, 20:80, 35:65, 50:50, 65:35, 80:20, 97:3] of ipsenol.

The second trapping experiment characterized the preference function of *I. avulsus* in terms of ipsdienol enantiomeric ratios. This experiment was conducted April-June 2010 and traps were baited with lanierone and racemic ipsenol and one of seven enantiomeric ratios [(+):(-): 3:97, 20:80, 35:65, 50:50, 65:35, 80:20, 97:3] of ipsdienol. Ipsenol was added to the lures to maximise capture, as demonstrated by Allison et al. (2012).

*Enantiomeric Ratio of Ipsdienol Produced by* I. avulsus *Adults. Ips avulsus* beetles were reared from naturally colonized loblolly pine, *Pinus taeda*, from the Kisatchie National Forest in Louisiana, USA (N31° 09.617’ W92° 40.117’). Beetles that emerged in a rearing enclosure (Browne 1972) were housed in vented plastic sample cups on moistened paper towels at 4° C. Beetles were sexed by the presence of the stridulatory apparatus on the heads of females, and males that were apparently healthy and active were released inside zippered pillow covers containing logs (ca. 15 cm diam. by ca. 65 cm long) from a healthy loblolly pine. These logs had been stored up to six weeks at 10° C following cutting of the tree. Approximately 50–100 drill holes (2 mm diam.) were spaced regularly around the bark surface to provide the beetles locations to begin mining and promote even spacing of attacks on the bark. After two days, 49 beetles were dissected from the bark and immediately frozen on dry ice. Their hindguts were then excised under isotonic saline solution and extracted by steeping for 0.5–2 h at room temperature in 50 µl redistilled hexane spiked with 3.8 ng/µl of cycloheptanone. Extracts (1 µl) were analyzed by coupled gas chromatography-mass spectrometry (Hewlett-Packard model G1800C, Palo Alto, CA, USA) with a chiral microcapillary column (β-dex 120; 30 m x 0.25 mm i.d. x 0.25 μm film thickness; Supelco Inc., Bellefonte, PA, USA) and with the temperature program 40° C for 1 min, 3° C/min to 145 °C, then 20 °C/min to 220 °C held 12 min. The mass spectral detector was operated in single ion monitoring mode. There was some overlap between the enantiomer peaks, which tailed significantly, and therefore standards composed of differing, known enantiomeric ratios of ipsdienol (purified enantiomers from Contech Inc., Burnaby, BC) were formulated and serially diluted to concentrations spanning the range of concentrations of ipsdienol in the samples. From the analyses of these standards, we constructed standard curves for converting the integration peak area ratios of ipsdienol in the samples to enantiomeric ratios.

*Statistical Analysis.* For each field experiment we tested for significant differences in captures among the different enantiomeric ratios tested. The number of captures did not follow a normal distribution, so data were analysed with non-parametric tests. We first used *Kruskal Wallis tests* (α = 0.05). We then used pairwise permutation tests (α = 0.05) as post hoc tests by using the coin package in R (Hothorn et al. 2006; R Core Team 2021). Because multiple tests were done on each dataset, P-values were corrected using a Bonferroni correction. Finally, we tested for a significant difference in variance between semiochemical production and response. Because the response data did not follow a normal distribution, we used a *Levene’s test* (α = 0.05) using the car package in R (Fox and Weisberg 2019).

## Results

I. avulsus *Preference Function.* In the first experiment, mean total captures by any single treatment ranged from 1052.25 ± 295.4 (mean ± SD) [97% (+)] to 1628.63 ± 502.9 [3% (+)] *I. avulsus* individuals (**Supplementary Table 1**). However, a significant treatment effect was not observed (*Kruskal-Wallis chi-squared* = 10.455, *df* = 6, P = 0.1067).

**Fig. 1:**
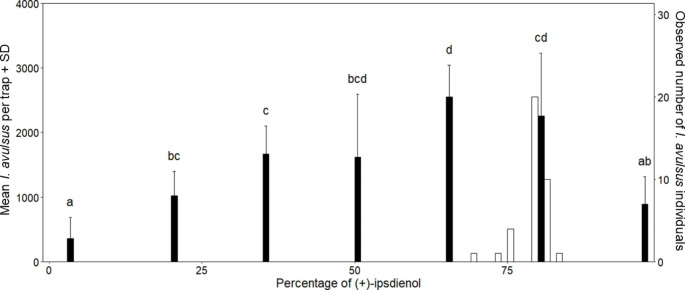
Distribution of (+)-ipsdienol enantiomeric ratios isolated from *I. avulsus* (white bars, right-hand axis) and mean number of *I. avulsus* individuals captured per trapping treatment (black bars, left-hand axis). Error bars represent standard deviation. Treatments marked with the same letter did not differ significantly (pairwise permutation tests; α = 0.05 with Bonferroni correction)

In the second experiment, mean total captures by any single treatment ranged from 355.5 ± 334.4 (mean ± SD) [3% (+)] to 2549.25 ± 495.7 [65% (+)] *I. avulsus* individuals (Fig. 1 and **Supplementary Table 2**). There was a significant difference among treatments (*Kruskal-Wallis chi-squared* = 33.604, *df* = 6, P < 0.001). On average, traps baited with 65% (+)-ipsdienol captured more *I. avulsus* than traps baited with the other treatments. However, no significant difference was found between traps baited with 65% (+)-ipsdienol and traps baited with 50% or 80% (+)-ipsdienol.

*Enantiomeric Ratio of Ipsdienol Produced by* I. avulsus *Adults. Ips avulsus* males produced an enantiomeric ratio of 79.4% ± 2.4 (mean ± SD) of (+)-ipsdienol (Fig. 1). Levene’s test (*F* = 62.231, *df* = 82,578, P < 0.001) indicated that variance in trap captures (SD = 24.27) was significantly greater than variance in production with regards to the enantiomeric ratio of ipsdienol. Signal production and preference function were both unimodal.

## Discussion

In this study we show that changes in the enantiomeric ratios of ipsdienol affected the capture of *I. avulsus* in the field (Fig. 1). Traps baited with lanierone, racemic ipsenol and 50%, 65% or 80% of (+)-ipsdienol captured the most beetles with no significant difference between trap captures by those three ratios (Fig. 1). On average, male *I. avulsus* produced an enantiomeric ratio of 79.4% of (+)-ipsdienol. This indicates that the optimum in the ratio of (+)-ipsdienol emitted by signalling individuals corresponds to the optimum in the ratio of (+)-ipsdienol to which male and female *I. avulsus* respond. Furthermore, this demonstrates that responding individuals respond indiscriminately to the different enantiomeric ratios produced by male *I. avulsus*. These observations match the predictions of the asymmetric tracking model proposed by Phelan (1992), a model developed based on contemporary understanding of the ecology of pheromone communication in moths. While the ecological traits and selective forces associated with bark beetle pheromone communication likely differ from the Lepidoptera, our study suggests that there might be similarities in the resulting interactions between the evolution of production and response traits.

Reported enantiomeric compositions of ipsdienol produced by male *I. avulsus* and inducing the strongest response from males and females have varied, suggesting geographic variation. Male *I. avulsus* produced an enantiomeric ratio of 90% of (+)-ipsdienol in East Texas (Kohnle et al. 1994) and an enantiomeric ratio of 75% of (+)-ipsdienol in Alabama (Seybold et al. 1995). In East Texas, Smith et al. (1990) found (-)-ipsdienol to be more attractive than (+)-ipsdienol and racemic ipsdienol, while Strom et al. (2003) found racemic ipsdienol to be more attractive than (+) and (-)-ipsdienol in Louisiana, Texas and Florida. Finally, Miller and Allison (2011) found racemic ipsdienol to be as attractive as (+)-ipsdienol in Georgia. This indicates that our observations are not inconsistent with previous reports. Indeed, population specific patterns of pheromone production and response have been demonstrated in other *Ips* species such as *Ips pini* (Lanier et al. 1980) and in *Ips subelongatus* (Song et al. 2011).

The main findings of this study are that female and male *I. avulsus* respond indiscriminately to the enantiomeric ratios produced by males and that the preference function exhibits more variance than the distribution of the pheromone signal in males. This corresponds to the predictions of the asymmetric tracking model (Phelan 1992). This model predicts that because sexes differ in terms of optimal strategy to maximize their own fitness, the response of the non-limiting sex will induce weak selective pressures onto the signal of the limiting sex. Emlen and Oring (1977) define the limiting sex as the sex that “provides the bulk of the parental investment”. Evolutionary changes in signal should rather be induced by stochastic factors and the necessity to avoid hybrid mating. The response trait in the non-limiting sex should evolve under the constraints of mate location and intrasexual competition. Ultimately, what Phelan (1992) describes is a great potential for sexual selection on the responding trait and a weaker, if not absent, potential for sexual selection on the emitting trait. This should induce the “tracking” behaviour of the signal trait by the response trait typical of the asymmetric tracking model. Indeed, because responding individuals have a broad preference function, a male that produces a divergent blend compared to the rest of the group would not see a proportional decline in reproductive success. If, because of other environmental factors, that male has a higher fitness than the average male, his phenotype will increase in frequency within the population. Consequently, the individuals that are, on average, more attracted to that pheromone blend are more likely to find a mate and a host. The frequency of their response phenotype will, in turn, increase in the population. Phelan (1992) predicted that this evolutionary relationship between signal and response should manifest as a greater variance in the response trait, regardless of the amount of variance observed in the signal.

Determining which of the sexes is the limiting sex in *I. avulsus* is slightly more challenging than in the Lepidoptera, mostly because of the high paternal reproductive investment. Cook et al. (1983) and Flamm et al. (1987) observed a 1:1 (male: female) sex ratio at re-emergence. They also acknowledged the evidence of multiple mating in some males, indicating that, in *I. avulsus*, the operational sex ratio must vary between 1:1 and a female-biased sex ratio depending on environmental conditions and time of the reproductive season. Additionally, Gouger et al. (1975) observed male *I. avulsus* blocking the entrance of the tunnel to potential mates, causing the females to try to force their way into the nuptial chamber while stridulating. Males likely use this intricate display for mate choice. The female biased operational sex ratio (Cook et al. 1983; Kirkendall 1983), even if temporary, along with male mate choice (Gouger et al. 1975; Clutton-Brock 2009) and spatially and temporally limited resource (Kirkendall 1983) could induce a great potential for sexual selection in females.

In responding males, the evolution of the response trait is also likely to be responding to sexual selection because their capacity to readily locate and colonise a new host influences their access to females and to available resources for their progeny. Consequently, like females, responding males would exhibit a great amount of variance in response. Once they have colonised a host and start emitting pheromone, the indiscriminate response by females will induce weak selective pressures onto the signal. The pheromone signal can instead evolve through stochastic events or under selection from external factors. These changes can then be tracked by male and female response. This pattern would allow for gradual evolution of the signal or for evolution through sudden saltational shifts as suggested by Roelofs et al. (2002) and Symonds and Elgar (2004).

One of the external factors that could create selective pressures and lead to changes in pheromone blends is the presence of related beetle species that use the same resource and the same compounds for pheromone communication. In Louisiana where these experiments were conducted, *I. avulsus*, *I calligraphus* and *I. grandicollis* are sympatric (Schoeller and Allison 2013). These species can even colonize hosts simultaneously (Paine et al. 1981) and can use the pheromone signal of a different species as a kairomone to locate ephemeral resource in the landscape. For example, *I. avulsus* responds to ipsenol, a pheromone compound of *I. grandicollis* (Allison et al. 2012). The necessity of resource partitioning and reproductive isolation could induce directional selection on the pheromone blends and would induce a shift in receiver preference function over time (Bacquet et al. 2015). For example, in the butterfly genus *Bicyclus*, sympatric species have achieved reproductive isolation through changes in male pheromones more rapidly than allopatric species (Bacquet et al. 2015).

The presence of predators could also induce changes in pheromone blends over time. Predatory beetles, particularly in the families Cleridae, Trogositidae, Histeridae and Cerambycidae, exploit bark beetle pheromone communication to find prey (Vité and Williamson 1970; Bakke and Kvamme 1981; Allison et al. 2001, 2013; Schoeller et al. 2012). Raffa and Klepzig (1989) demonstrated that the predator *Thanasimus dubius* responds to a different enantiomeric ratio of ipsdienol than its prey *I. pini*. They hypothesized that the pheromone blend of *I. pini* is evolving as a response to selective pressures induced by the presence of the predator (Raffa and Dahlsten 1995; Shumate et al. 2011).

The biosynthetic pathways involved in pheromone synthesis in *I. avulsus* might prevent the appearance of radically different signals. In the *Ips* genus, ipsdienol seems to be synthesized *de novo* as well as from the host precursor, myrcene (Blomquist et al. 2010). While the de novo pathway could evolve by responding to selective pressures acting on the bark beetles, the pathway that uses host precursor is limited in its response to selection and can dictate the identity and profile of the pheromone signal in *Ips* species (Lindström et al. 1989).

Another factor that would affect the evolution of pheromone blends over time are symbiotic microorganisms. Bark beetles have symbiotic fungi that enable the colonization of the host and the digestion of wood (Harrington 2005). They also carry a gut microbiome. These microorganisms participate in the production of the compounds used as aggregation pheromones in bark beetles (Byers and Wood 1981; Hunt and Borden 1989; Zhao et al. 2015). It has even been hypothesized that some of these symbionts use those compounds to attract host beetles (Zhao et al. 2019). If the communication system between the fungus and the beetles also relies on those same compounds, the symbiosis would induce stabilizing selection on the receiver preference function. Additionally, through horizontal transfers via trophic and intraspecific interactions, or through maternal inheritance, symbiotic microorganisms often have different inheritance patterns than the nuclear genes coding for pheromone production. This can slow down response to selection (Zeh and Zeh 2005).

In this study we demonstrated that the enantiomeric ratio of ipsdienol affects trap captures of *I. avulsus*. Traps baited with 50%, 65% and 80% of (+)-ipsdienol were most efficient at capturing *I. avulsus* beetles. Our results also show that the evolution of the aggregation pheromone produced by male *I. avulsus* is not being constrained by the response of conspecifics, as suggested by the asymmetric model. We hypothesize that the evolution of these pheromone blends might be subjected to external selection forces such as predation, the response of other beetle species to the pheromone blends or the interaction between the bark beetles and symbiotic microorganisms. Our study joins a body of literature advocating for further research into bark beetle reproductive ecology (Kirkendall 1983; Symonds and Elgar 2004; Shumate et al. 2011) as it has become evident that their complex ecology deeply impacts pheromone evolution in these species.

## Electronic Supplementary Material

Below is the link to the electronic supplementary material.

Supplementary Material 1

Supplementary Material 2

Supplementary Material 3
