# Supplementary material for: No Evidence of Responding Individuals Constraining the Evolution of the Pheromone Signal in the Pine Engraver Ips avulsus
Source: J Chem Ecol. 2022 Dec 10;49(1-2):11–7. doi: 10.1007/s10886-022-01396-w (PMC9941236; doi:10.1007/s10886-022-01396-w)
Supplement: Supplementary file 1 — Supplementary Material 1 [file 10886_2022_1396_MOESM1_ESM.docx]

| **Supplementary table 1** Number of *I. avulsus* beetles captured by individual traps bated with lanierone, racemic ipsdienol and one of seven enantiomeric ratios of (+)-ipsenol. | | | |
| --- | --- | --- | --- |
| Percentage of (+)-ipsenol in the lure | Trap number | Captures | Mean ± SD |
| 3 | 6 | 1714 |  |
|  | 8 | 966 |  |
|  | 18 | 1033 |  |
|  | 24 | 2363 |  |
|  | 31 | 1737 |  |
|  | 36 | 2203 |  |
|  | 47 | 1335 |  |
|  | 53 | 1678 | 1628.6 ± 502.9 |
| 20 | 7 | 1507 |  |
|  | 9 | 1278 |  |
|  | 15 | 1273 |  |
|  | 23 | 1442 |  |
|  | 33 | 1688 |  |
|  | 41 | 2180 |  |
|  | 49 | 1411 |  |
|  | 52 | 1611 | 1548.8 ± 293.5 |
| 35 | 3 | 1760 |  |
|  | 11 | 942 |  |
|  | 21 | 1046 |  |
|  | 27 | 1464 |  |
|  | 34 | 1271 |  |
|  | 39 | 3043 |  |
|  | 46 | 1633 |  |
|  | 54 | 1231 | 1548.8 ± 664.5 |
| 50 | 5 | 1641 |  |
|  | 10 | 715 |  |
|  | 19 | 1296 |  |
|  | 26 | 1021 |  |
|  | 32 | 1109 |  |
|  | 42 | 3092 |  |
|  | 45 | 1678 |  |
|  | 55 | 1237 | 1473.6 ± 726.0 |
| 65 | 1 | 1627 |  |
|  | 13 | 1184 |  |
|  | 17 | 815 |  |
|  | 28 | 1037 |  |
|  | 35 | 1402 |  |
|  | 37 | 2002 |  |
|  | 43 | 1975 |  |
|  | 50 | 1524 | 1445.8 ± 425.0 |
| 80 | 4 | 1526 |  |
|  | 12 | 1143 |  |
|  | 16 | 475 |  |
|  | 22 | 1472 |  |
|  | 30 | 1147 |  |
|  | 38 | 986 |  |
|  | 44 | 1594 |  |
|  | 51 | 1209 | 1194 ± 361.1 |
| 97 | 2 | 1320 |  |
|  | 14 | 1269 |  |
|  | 20 | 978 |  |
|  | 25 | 625 |  |
|  | 29 | 1020 |  |
|  | 40 | 1465 |  |
|  | 48 | 1053 |  |
|  | 56 | 688 | 1052.3 ± 295.4 |
