# Supplementary material for: No Evidence of Responding Individuals Constraining the Evolution of the Pheromone Signal in the Pine Engraver Ips avulsus
Source: J Chem Ecol. 2022 Dec 10;49(1-2):11–7. doi: 10.1007/s10886-022-01396-w (PMC9941236; doi:10.1007/s10886-022-01396-w)
Supplement: Supplementary file 2 — Supplementary Material 2 [file 10886_2022_1396_MOESM2_ESM.docx]

| **Supplementary table 2** Number of *I. avulsus* beetles captured by individual traps bated with lanierone, racemic ipsenol and one of seven enantiomeric ratios of (+)-ipsdienol. | | | |
| --- | --- | --- | --- |
| Percentage of (+)-ipsdienol in the lure | Trap number | Captures | Mean ± SD |
| 3 | 3 | 88 |  |
|  | 8 | 648 |  |
|  | 17 | 189 |  |
|  | 22 | 123 |  |
|  | 31 | 174 |  |
|  | 36 | 140 |  |
|  | 44 | 1030 |  |
|  | 55 | 452 | 355.5 ± 334.4 |
| 20 | 1 | 1185 |  |
|  | 12 | 739 |  |
|  | 20 | 1047 |  |
|  | 23 | 1004 |  |
|  | 33 | 1196 |  |
|  | 40 | 1537 |  |
|  | 45 | 1213 |  |
|  | 56 | 285 | 1025.8 ± 374.7 |
| 35 | 2 | 1937 |  |
|  | 9 | 2419 |  |
|  | 21 | 1532 |  |
|  | 24 | 1777 |  |
|  | 32 | 1620 |  |
|  | 37 | 1814 |  |
|  | 48 | 1253 |  |
|  | 54 | 1007 | 1669.9 ± 430.9 |
| 50 | 5 | 208 |  |
|  | 11 | 166 |  |
|  | 19 | 1321 |  |
|  | 26 | 2069 |  |
|  | 35 | 2268 |  |
|  | 42 | 2084 |  |
|  | 49 | 2063 |  |
|  | 51 | 2796 | 1621.9 ± 971.9 |
| 65 | 7 | 2981 |  |
|  | 10 | 2797 |  |
|  | 15 | 2554 |  |
|  | 25 | 2057 |  |
|  | 34 | 3312 |  |
|  | 41 | 1781 |  |
|  | 43 | 2598 |  |
|  | 52 | 2314 | 2549.3 ± 495.7 |
| 80 | 6 | 3172 |  |
|  | 13 | 420 |  |
|  | 18 | 3098 |  |
|  | 27 | 1999 |  |
|  | 29 | 1510 |  |
|  | 38 | 2411 |  |
|  | 47 | 2121 |  |
|  | 50 | 3299 | 2253.8 ± 976.3 |
| 97 | 4 | 383 |  |
|  | 14 | 569 |  |
|  | 16 | 583 |  |
|  | 28 | 773 |  |
|  | 30 | 1200 |  |
|  | 39 | 843 |  |
|  | 46 | 1712 |  |
|  | 53 | 1060 | 890.4 ± 426.1 |
