# Supplementary material for: No Evidence of Responding Individuals Constraining the Evolution of the Pheromone Signal in the Pine Engraver Ips avulsus
Source: J Chem Ecol. 2022 Dec 10;49(1-2):11–7. doi: 10.1007/s10886-022-01396-w (PMC9941236; doi:10.1007/s10886-022-01396-w)
Supplement: Supplementary file 3 — Supplementary Material 3 [file 10886_2022_1396_MOESM3_ESM.docx]

SUPPLEMENTARY MATERIALS

The enantioselective column did not provide complete resolution of ipsdienol enantiomers, even after extensive adjustment to the method and replacement of the original column with an unused one (best program: 3°/min from 40°; 0.65 ml/min flow). Additionally, the peaks were tailing significantly.

45.3%%

54.7%

Retention time

Ion abundance

**Supplementary fig. 1** Example of an analysis of racemic ipsdienol using the optimal GC settings.

Integration software is commonly used to separate overlapping peaks either by dropping a vertical line from the lowest point of the valley between peaks to the baseline (baseline drop method) or by setting the baseline to connect the lowest point of the valley to the baseline on either side of the overlapping peaks (valley-valley method). In this study, the integration parameters possible on the integration software available to us were not capable of providing reliable quantitation of the overlapping peaks. The issue was further complicated by peak tailing (Meyer 1995). With both baseline drop and valley-valley methods, we found that the ratio of integration areas of two overlapping peaks did not maintain a linear relationship with the ratio between the associated compounds in the sample, and that this relationship was also dependent on peak heights (i.e., sample concentration).

To address this issue, we constructed “two-dimensional” quantitative standards to allow accurate calculation of percentage of (+)-ipsdienol in *I. avulsus* samples*.* These standards varied both in the percentage of (+)-ipsdienol and in concentration. Our preliminary approximation of the mean enantiomeric composition for *I. avulsus* was 80% (+)-ipsdienol with up to 5% of variation around this value. Therefore, we formulated three standards with 75%, 80%, and 85% of (+)-ipsdienol respectively. We expected this range would enclose all beetle samples, except for a single outlier. Each of the three standards were analyzed at four concentrations that we expected would encompass the range of concentrations of ipsdienol in the beetle samples. For each percentage of (+)-ipsdienol injected (i.e., 75%, 80%, 85%), there was a strong linear relationship between the log of the integration area of the (-)-ipsdienol peak (proxy for quantity injected) and the percentage represented by integration area of the (+)-ipsdienol peak (**Supplementary fig. 2**).

**Supplementary fig. 2** Distribution of the integration areas measured for each *I. avulsus* male sampled (purple crosses) and linear regressions (colored lines) between the integration area of the (+)- ipsdienol peak and the log 10 of the integration area of the (-)-ipsdienol peak within each of the three tested standards. The blue diamonds represent the measurements of the 75% (+)-ipsdienol standard, the red squares represent the measurements of the 80% (+)-ipsdienol standard, and the green triangles represent the measurements of the 85% (+)-ipsdienol standard.

Furthermore, for each of the four concentrations tested, the relationship between percentages of enantiomers in the standards and percentages of integration areas of the respective enantiomer peaks was linear (**Supplementary fig. 3**).

**Supplementary fig. 3** Linear regression between the percentage of integration area of the (+)-ipsdienol peak and the percentage of (+)-ipsdienol in the standard. The different colored circles indicate one of four different concentrations at which the standards were analyzed.

**Supplementary fig. 4** Graphical representation of the correction used to calculate the enantiomeric compositions of the ipsdienol sampled from *I. avulsus* males. A similar strategy was used for data points between the 80% and 85% (+)-ipsdienol standards.

For each sample, Y values for the intercept of the integration area for (-)-ipsdienol in the sample and the regression line for each standard (i.e., those shown in **Supplementary fig. 2**) were calculated. If the percentage of the integration area (Y_S_) of the (+)-ipsdienol peak for a sample fell between two of these Y values (e.g., Y_75%_ and Y_80%_), the proportion of (+)-ipsdienol in the sample was calculated as (Y_S_ - Y_75%_)/(Y_80%_ - Y_75%_)*5% + 75%. This was done similarly when Y_S_ fell between Y_80%_ and Y_85%,_ but with 80% replacing 75% in the calculation. In the case of the outlier (Y_S_ was considerably lower than Y_75%_), the enantiomeric ratio was interpolated using Y_75%_ and Y_80%_ for the calculation. Based on the residuals of the regression lines of the standards in **Supplementary fig. 2**, we estimate the individual measurements of percentage of (+)-ipsdienol in the samples were accurate within 1%.

REFERENCES

Meyer VR (1995) Errors in the area determination of incompletely resolved chromatographic peaks. Journal of Chromatographic Science 33:26–33. https://doi.org/10.1093/chromsci/33.1.26
